# Comparison of HER-2 overexpression in primary breast cancer and metastatic sites and its effect on biological targeting therapy of metastatic disease

**DOI:** 10.1038/sj.bjc.6602738

**Published:** 2005-08-16

**Authors:** J Zidan, I Dashkovsky, C Stayerman, W Basher, C Cozacov, A Hadary

**Affiliations:** 1 Oncology Unit, Sieff Government Hospital, Safed, Israel; 2 Faculty of Medicine, Israel Institute of Technology (Technion), Haifa, Israel; 3 Department of Surgery, Sieff Government Hospital, Safed, Israel; 4 Department of Pathology, Sieff Government Hospital, Safed, Israel

**Keywords:** comparison, HER-2 overexpression, metastases, primary breast cancer, treatment

## Abstract

HER-2 overexpression, a predictive marker of tumour aggressiveness and responsiveness to therapy, occurs in 20–30% of breast cancer. Although breast cancer is a heterogeneous disease, HER-2 measurement is carried out in primary tumour. This study aims to evaluate HER-2 overexpression in primary and metastases and its effect on treatment decisions. Biopsies from primary breast cancer and corresponding metastases from 58 patients were studied. HER-2 overexpression was evaluated immunohistochemically in all primary and metastatic sites. Positive overexpression in primary and/or metastases was confirmed by fluorescence *in situ* hybridisation (FISH). Discordance in HER-2 overexpression between primary and metastatic sites was 14% (eight of 58 patients). Concordance was found in 50 (86%) of patients (95% CI: 77–95). In one patient (2%), HER-2 was negative in metastasis but positive in primary. In seven (12%) patients, HER-2 was positive in metastases and negative in primary (95% CI: 3.7–20), and three of them responded to trastuzumab. Gene amplification by FISH was found in all cases with HER-2 positive (+2 and +3) by immunohistochemistry. Our data suggest that a possible discordance of HER-2 overexpression between primary and metastases should be considered when making treatment decisions in patients with primary HER-2-negative tumours.

The choice of therapy for treatment of breast cancer is based on tumour stage, histopathologic features, hormone receptor status and biological markers (Hamilton and Piccart, 2000). The most promising biological marker in terms of predicative value for breast cancer treatment is HER-2 (Thor *et al*, 1998). The HER-2 oncoprotein is a transmembrane receptor, belonging to the epidermal growth factor receptor family, with tyrosine kinase activity, resulting in intracellular signalling and activation of genes involved in cell growth, which is associated with shortened survival, enhanced aggressiveness and other poor prognostic factors (Slamon *et al*, 1987; Tsuda *et al*, 1989). Most of the literature reports a correlation between HER-2 overexpression and increased sensitivity to anthracycline-based chemotherapy in comparison with CMF and tamoxifen (Elledge *et al*, 1998; Paik *et al*, 1998). Most importantly, HER-2 overexpression identifies the subset of patients who can benefit from trastuzumab, recombinant humanised anti-HER-2 monoclonal antibody (Genentech Inc., South San Francisco, CA, USA), in advanced and metastatic breast cancer (MBC) (Hortobagyi, 2001; Lohrisch and Piccart, 2001). Monotherapy with trastuzumab has yielded response rates of 35% when given as first-line therapy and 18% in patients previously treated with chemotherapy (Cobleigh, 1999; Vogel *et al*, 2001). Compared with chemotherapy alone, combination of chemotherapy and trastuzumab improves response rates and overall survival for women with HER-2-positive MBC (Lohrisch and Piccart, 2001). Breast cancer is a heterogeneous tumour with high individual variability as far as response to treatment is concerned (Harris *et al*, 1997).

Generally, assessment of HER-2 is performed in the primary tumour even if the metastases appear several years later. As biopsy of metastases is not routine, it is difficult to test a large number of cases. Clinical metastases may grow from micrometastases resistant to adjuvant therapy and may represent one particularly aggressive clone from among many clones of primary breast cancer (Harris *et al*, 1997). It is still unknown whether HER-2 expression differs in metastases compared to primary breast cancer. Few data have been published regarding this issue (Cardoso *et al*, 2001).

The objective of the present study is to determine the expression of HER-2 in the primary breast cancer and its metastases and to update our previous results by studying additional patients (Zidan *et al*, 2002). All tumours and their metastases were evaluated for HER-2 using immunohistochemistry (IHC) (Hercept Test). The fluorescence *in situ* hybridisation (FISH) test was utilised for HER-2-overexpressing cases. In addition, we examined the utility of evaluating HER-2 overexpression in metastases on the decision to treat these patients with trastuzumab. A comprehensive review of the literature was also performed.

## MATERIALS AND METHODS

Between 1990 and 2002, 209 patients with MBC were treated in our department. Patients were enrolled in this study if tumour samples from both primary and corresponding metastases were available and suitable for IHC analysis. Patients with metastatic ipsilateral axillary lymph nodes at the primary operation were excluded.

Formalin-fixed paraffin-embedded samples of primary breast cancer and metastases were collected from 58 patients. For each case, the primary tumour and the corresponding metastases were sampled for HER-2 staining at the same time by the same two experienced pathologists. Immunohistochemical staining for HER-2 was performed by using CB11 monoclonal antibody with antigen retrieval. Expression was scored using the scoring system outlined in the DAKO Hercept Test as 0, 1+, 2+ and 3+ according to the standardised criteria (Slamon *et al*, 1989). Gene amplification by FISH was tested in all cases where the primary tumour or metastases were positive (+2 or +3) by IHC.

### Statistical analysis

Rates and positivity were compared using McNamara tests for paired data. Confidence intervals for concordance/discordance rates were calculated. The Kendall's tau-*b* coefficient was used to assess concordance for HER-2 status. Although the difference between HER-2 overexpression in the primary tumour and the metastases was marginally significant (*P*=0.07), a discordance was observed (Kendall's tau-*b* coefficient=0.69, 95% CI=0.53–0.80) due to more cases of HER-2 overexpression in the metastases. Two-tailed *P*-values of 0.05 or less was considered to have a statistical significance.

## RESULTS

All 58 cases with available biopsies from the primary tumour and metastases were included in this study. Median age at diagnosis of the primary breast cancer was 56 years (range: 29–82 years). One patient was male. Metastases were found in bone of 68% of patients (*n*=39), in skin and soft tissue in the surgical scar region of 35% (*n*=20), in 36% (*n*=21) in liver, in lungs of 33% (*n*=19) of patients and in the pleura of 19% (*n*=11) of patients. In 35% (*n*=20) of patients, there was one metastatic site, in 41% (*n*=24) of patients two sites were found and in 24% (*n*=14) three metastases were found (Table 1). Invasive ductal carcinoma was diagnosed in 88% of patients. Oestrogen and progesterone receptors (ER, PR) were positive in 60 and 53% of the primary tumours, respectively. All patients underwent surgery at diagnosis: 60% (*n*=35) of patients had lumpectomy and 40% (*n*=23) had mastectomy. In all, 29 (50%) patients were treated with tamoxifen as adjuvant treatment with or without chemotherapy.

Metastases were diagnosed between 1 and 12 years after primary breast cancer surgery (median: 4.5 years). HER-2 was positive by IHC in 24% (*n*=14) of the primary breast cancer tissues (two with 2+ and 12 with 3+) and negative in the remaining 76% (*n*=44). In 35% (*n*=20) of patients, HER-2 was positive in metastases (two with 2+ and 18 with 3+). Of the total 58 patients, 86% (*n*=50) revealed a concordance between the primary tumour and its metastases (95% CI: 77–95). In 2% of patients (*n*=1), HER-2 was positive in the primary tumour and negative in the metastatic site. In 12% (*n*=7) of patients, HER-2 was negative in the primary tumour and positive in the corresponding metastases (95% CI: 3, 7–20) (*P*=0.07) (Table 2). The single patient with HER-2 positive in primary carcinoma of breast and negative in skin and bone metastases was a 48-year-old woman in whom metastases were diagnosed 2 years postmastectomy. Trastuzumab and vinorelbine were given as second-line therapy for 3 months then stopped because of disease progression.

Four patients were treated with trastuzumab due to HER-2 evaluation in the metastases as follows:

*Case 1*: A 32-year-old male patient had metastases in skin, bone and lung 3 years after mastectomy. HER-2 was negative in the primary tumour but positive in skin and bone metastases. After failure with doxorubicin and paclitaxel, he received trastuzumab as a single agent and achieved partial response for 9 months.

*Case 2*: A 58-year-old woman with primary HER-2 negative but positive in metastases in liver (after 6 years) received trastuzumab monotherapy as third-line therapy after chemotherapy failure for 6 months with minimal response and no side effects.

*Case 3*: A 61-year-old woman had a local recurrence in skin and soft tissue of chest wall 5 years after mastectomy. Oestrogen receptor and PR were positive. She received tamoxifen as an adjuvant treatment for 5 years. At the end of the 5th year, she was diagnosed with multiple local recurrences in skin and soft tissue. HER-2 was negative in the primary and positive (+2) in the recurrence. Gene amplification by FISH was negative in the primary and positive in the recurrence. ER and PR were found to be positive in the recurrence as well. After chemotherapy failure (two lines), the patient received trastuzumab with vinorelbine weekly, achieving complete response after 5 months. For the past 13 months, this patient has received only trastuzumab and still in complete remission with high quality of life.

*Case 4*: A 52–year-old woman with lung metastases with HER-2 negative in primary and positive in metastases did not respond to trastuzumab therapy. Three deceased patients who had not received trastuzumab were studied retrospectively.

## DISCUSSION

The most promising predictive factor for breast cancer today is HER-2. Breast tumours with HER-2 overexpression are resistant to hormonal therapy and alkylating agents, but more sensitive to anthracycline-based chemotherapy (Elledge *et al*, 1998; Ross and Fletcher, 1998; Hamilton and Piccart, 2000). Studies that have addressed HER-2 as a predictive factor for hormonal therapy have demonstrated lower response rates or reduced survival in hormonally treated patients whose tumours overexpressed HER-2 (level 2+ or 3+), reaching statistical significance in most studies (Nicholson *et al*, 1993; Leitzel *et al*, 1995; Elledge *et al*, 1998).

HER-2 overexpression is gaining acceptance as an indicator that defines breast cancer patients who should preferentially receive anthracycline-based or CMF adjuvant or therapeutic treatment (Paik *et al*, 1998; Thor *et al*, 1998; Hamilton and Piccart, 2000; Andrulis *et al*, 1998). HER-2 overexpression is a prerequisite for treating patients with MBC with trastuzumab, and may be considered for adjuvant treatment in the future (Ross and Fletcher, 1998; Andrulis, 2001; Eiermann, 2001).

Generally, evaluation of HER-2 overexpression for the treatment of MBC is carried out in the primary tumour. Overexpression is shown in 20–77% of human breast cancers (Slichenmyer and Fry, 2001). Modern diagnostic techniques and tumour markers can detect metastases without the need for routine biopsy of metastases. On the other hand, axillary lymph node dissection is a standard procedure of surgical treatment of breast cancer. This makes the evaluation of HER-2 in metastases difficult compared to primary breast cancer and axillary lymph nodes. Most studies occurred over a period of years because of the difficulty in recruiting patients (Gancberg *et al*, 2002). Cardoso *et al* (2001) evaluated HER-2 overexpression in primary breast cancer and metastatic axillary lymph nodes in 370 patients. HER-2 was positive in 10% of the primary and 11% of the metastases. In all, 98% of these patients revealed a concordance between primary tumour and lymph nodes.

Distant metastases should not be assumed to be biologically equivalent to lymph node metastases. Distant metastases are generally identified years after resection of the primary cancer and axillary lymph nodes, and represent clonal outgrowths with genetic modifications not detected in the primary tumour. Additionally, cells that metastasise via the lymphatic system may possess biological properties that permit travel to distant sites and establishment of clinically disseminated disease (Harris *et al*, 1997).

The present study suggests a 14% discordance of HER-2 overexpression between primary tumour and the metastases (*P*=0.07 McNamara). HER-2 was positive in the primary and negative in the metastatic site in one patient (2%) and negative in the primary and positive in metastases in seven patients (12%). In these seven patients, findings affected the selection of therapy, particularly by trastuzumab, in four who otherwise would not have been selected for trastuzumab treatment.

A review of the literature found only 11 studies comparing HER-2 overexpression in primary breast cancer and metastases (Table 3). These studies show discordance rates of between 0 and 26%. Niehans *et al* (1993) retrospectively evaluated HER-2 overexpression in primary breast cancer and different metastatic sites in autopsy tumour samples. The uncontrolled fixation time of autopsy samples jeopardises the IHC data that can be obtained from samples. Masood and Bui (2000) evaluated HER-2 overexpression in 56 patients, but only 11 cases of metastases were from distant sites. At the 2000 San Antonio Breast Cancer Meeting, Edgerton *et al* (2000) presented preliminary results of a study comparing HER-2 status in 193 patients, only 93 of which were distant metastases. Only seven of the 21 metastatic samples evaluated by Shimizu *et al* (2000) were from distant sites. Tanner *et al* (2001) reported on 46 primary breast cancers and their metastases. Only 12 of the 46 cases were metastases from distant sites. Gancberg *et al* (2002) utilised both IHC and FISH techniques retrospectively in 107 patients between 1991 and 1999. Only 68 out of the 107 cases were available for FISH test and five (7%) of them were discordant. In our previous study, 40 patients with primary and metastases were evaluated by IHC test. Discordance was found in 18% of cases (Zidan *et al*, 2002) and in 14% when 58 patients were evaluated using both IHC and FISH (Zidan *et al*, 2004). Dowsett *et al* (2003) reported on 8% discordance. In the study presented by Lipton *et al* (2003), serum was obtained at the time of disease progression from 240 patients who were initially serum HER-2 negative at the start of hormone therapy. In 62 (26%) of 240 patients, serum HER-2 converted from negative to positive at the time of disease progression. Luftner *et al* (2004) found discordance in 18% of 80 cases using IHC. An analysis of the studies summarised in Table 3 shows that the seven studies with the largest number of patients found discordance to be ⩾7 to 26% and four studies showed a discordance rate of 0–3%. These four studies were actually based on small numbers of distant metastases and include mostly locoregional relapses.

Immunohistochemistry staining has certain limitations, this cannot, however, explain the discordance observed between primary and metastases in the expression of HER-2 in the present study. The same IHC method was used for all patients and evaluation was carried out by the same pathologist. Primary and metastatic lesions from the same patients were immunostained in the same staining run, thus the probability of error is minimised.

The best method of measuring HER-2 is a point of controversy. A consensus conference was held in August 1999 in an attempt to resolve some of these issues (Author unlisted, 1999). Data presented at that meeting indicate that the concordance between IHC and FISH methods is in the range of 80–90%, comparing 3+ overexpression. As the median time between primary tumour and metastases in our study is 4.5 years, it is difficult to compare HER-2 overexpression by using frozen tissue samples from both primary and metastases. This technical limitation may affect the results of IHC test for HER-2 expression in some cases. FISH technique was used for testing and confirming all cases with HER-2 overexpression by IHC (+2 and +3). All cases in our study showed amplification by FISH. The first limitation of FISH is the much higher cost when compared to IHC test. The second limitation is that, although IHC can be carried out in nearly every pathology laboratory, FISH can be performed only in special laboratories with specialised equipment.

The present study is one of the first to address the issue of heterogeneity between breast cancer primaries and metastatic sites. The rationale behind the present study is the fact that breast cancer is a biologically and genetically heterogeneous tumour that contains multiple different clones (Teixeira *et al*, 1995). An early stem line clone may migrate to the metastatic site and grow independently from its counterpart in the primary tumour, resulting in complete heterogeneity between primary and metastatic sites in the same patient. Heterogeneity between primary and metastatic sites may also result from the genetic instability of cancer cells. Breast cancer cells that survive adjuvant treatment may undergo genetic changes resulting in either a loss or gain of expression of some biological markers (Kuukasjarvi *et al*, 1997). Molecular changes, including upregulation of HER-2, were observed in breast cancer relapsing after adjuvant hormone therapy (Slichenmyer and Fry, 2001; Tanner *et al*, 2001). In our study, four of seven patients with HER-2 positive in metastases and negative in the primary initially received adjuvant treatment with tamoxifen before developing metastases. Two became ER negative in metastases. Additionally, some small clones in the primary tumour overexpress HER-2, but are negligible in the tumour mass and may not be detected in the staining of the larger primary breast cancer. Metastases developing from such clones are HER-2 positive (Teixeira *et al*, 1995).

In all, 12% of patients in the current study were found to be eligible for treatment with trastuzumab, according to results of HER-2 overexpression in metastatic sites. To our knowledge, ours is the first study to address the possibility of treating patients with metastases of breast cancer with trastuzumab based on the discordance in HER-2 overexpression between primary tumour and metastases.

Three other techniques appear easy and applicable in evaluating HER-2 overexpression in MBC. One promising method is the testing of extracellular domain of HER-2 in serum of breast cancer patients (Burstein *et al*, 2003). Another technique is the evaluation of HER-2 amplification by chromogenic *in situ* hybridisation. This technique is easy, inexpensive and can be carried out in most hospitals (Dandachi *et al*, 2004). HER-2 amplification detected by FISH in fine-needle aspiration (FNA) from metastases is also a promising technology. Bozzetti *et al* (2002) evaluate HER-2 amplification by FISH on 66 breast cancer FNAs. Paired results by FISH cytology and FISH histology showed a concordance of 91%. They concluded that HER-2 gene amplification can be reliably estimated by FISH on breast cancer FNAs. This makes the evaluation of HER-2 from metastases easy and implementable.

To summarise, the present study demonstrates a relatively high discordance rate (14%) in HER-2 overexpression between primary and metastases of the same breast cancer, emphasising the existence of biological differences between primary and metastases. We suggest taking HER-2 evaluation into consideration in metastatic sites when HER-2 is negative in the primary tumour and the patient can benefit from treatment with trastuzumab. Additional studies with larger numbers of patients are needed to verify our results.

## Figures and Tables

**Table 1:** Patient characteristics

	**Number of patients**	**%**
Total number of patients	58	100
		
*Gender*		
Female	57	98
Male	1	2
		
*Age (years)*		
Median	56	
Range	29–82	
		
*Receptor status, primary tumour*		
ER positive	35	60
PR positive	31	53
		
*Primary surgery*		
Lumpectomy	35	60
Mastectomy	23	40
		
*Histology*		
Invasive ductal carcinoma	51	88
Invasive lobular carcinoma	7	12
		
*Number of metastatic sites*		
1	20	35
2	24	41
3	14	24
		
*Prior therapy*		
Adjuvant chemotherapy	29	50
Adjuvant hormonal therapy	10	18
Adjuvant chemo+hormonal therapy	19	32

ER=oestrogen receptor; PR=progesterone receptor.

**Table 2:** Comparison of 21 patients with HER-2 positive in primary breast cancer and/or metastatic site

**Case #**	**HER-2 (IHC) in primary**	**HER-2 (FISH) in primary**	**Metastatic site**	**HER-2 (IHC) in metastases**	**HER-2 (FISH) in metastases**
1	+3	Positive	Skin, soft tissue	+3	Positive
2	+3	Positive	Skin, bone	+3	Positive
3	+3	Positive	Local recurrence in breast	+3	Positive
4	+3	Positive	Pleura	+3	Positive
5	+3	Positive	Liver	+3	Positive
6	+3	Positive	Liver	+3	Positive
7	+2	Positive	Lung	+3	Positive
8	+3	Positive	Skin	+3	Positive
9	+3	Positive	Bone	+2	Positive
10	+3	Positive	Lung	+3	Positive
11	+2	Positive	Bone	+3	Positive
12	+3	Positive	Skin	+3	Positive
13	+3	Positive	Liver	+3	Positive
14	+3	Positive	Skin and bone	Negative	Negative
15	Negative	Negative	Skin, soft tissue	+3	Positive
16	Negative	Negative	Soft tissue, bone	+3	Positive
17	Negative	Negative	Skin, bone, lung	+3	Positive
18	Negative	Negative	Liver	+3	Positive
19	Negative	Negative	Skin, soft tissue	+2	Positive
20	Negative	Negative	Pleura	+3	Positive
21	Negative	Negative	Lung	+3	Positive

IHC=immunohistochemistry; FISH=fluorescence *in situ* hybridisation.

**Table 3:** Studies comparing HER-2 overexpression in primary breast cancer and distant metastases

**Investigator**	**# of pts.**	**HER-2 overexpression: evaluation method**	**Discordance (%) in HER-2 between primary and metastasis**
Neihans	30	IHC	3
Masood	56	IHC	2
Edgerton	193	IHC and FISH	25
Shimizu	21	IHC	0
Tanner	46	IHC and CISH	0
Gancberg	107	IHC and FISH	7
Zidan	40	IHC	18
Zidan	58	IHC and FISH	14
Dowsett	39	IHC	8
Lipton	240	Serum ECD	26
Luftner	80	IHC	18

IHC=immunohistochemistry; FISH=fluorescence *in situ* hybridisation; CISH=chromogenic *in situ* hybridisation; ECD=extracellular domain.

## References

[bib1] Andrulis IL, Bull SB, Blackstein ME, Sutherland D, Mak C, Sidlofsky S, Pritzker KP, Hartwick RW, Hanna W, Lickley L, Wilkinson R, Qizilbash A, Ambus U, Lipa M, Weizel H, Katz A, Baida M, Mariz S, Stoik G, Dacamara P, Strongitharm D, Geddie W, McCready D (1998) Neu/erbB-2 amplification identifies a poor-prognosis group of women with node-negative breast cancer. Toronto Breast Cancer Study Group. J Clin Oncol 16(4): 1340–1349 9552035 10.1200/JCO.1998.16.4.1340

[bib2] Baselga J (2001) Phase I and II clinical trials of trastuzumab. Ann Oncol 12(Suppl 1): 49–55 10.1093/annonc/12.suppl_1.s49 11521722

[bib3] Bozzetti C, Nizzoli R, Guazzi A, Flora M, Bassano C, Crafa P, Naldi N, Cascinu S (2002) HER-2/neu amplification detected by fluorescence *in situ* hybridization in fine needle aspirates from primary breast cancer. Ann Oncol 13: 1398–1403 12196365 10.1093/annonc/mdf217

[bib4] Burstein HJ, Harris LN, Marcom PK, Lambert-Falls R, Havlin K, Overmoyer B, Friedlander Jr RJ, Gargiulo J, Strenger R, Vogel CL, Ryan PD, Ellis MJ, Nunes RA, Bunnell CA, Campos SM, Hallor M, Gelman R, Winer EP (2003) Trastuzumab and vinorelbine as first-line therapy for HER2-overexpressing metastatic breast cancer: multicenter phase II trial with clinical outcomes, analysis of serum tumor markers as predictive factors, and cardiac surveillance algorithm. J Clin Oncol 21: 2889–2895 12885806 10.1200/JCO.2003.02.018

[bib5] Cardoso F, Di Leo A, Larsimont D, Gancberg D, Rouas G, Dolci S, Ferreira F, Paesmans M, Piccart M (2001) Evaluation of HER2, p53, bcl-2, topoisomerase II-alpha, heat shock proteins 27 and 70 in primary breast cancer and metastatic ipsilateral axillary lymph nodes. Ann Oncol 12: 615–620 11432618 10.1023/a:1011182524684

[bib6] Cobleigh M, Vogel CH, Tripathy D (1999) Multinational study of the efficacy and safety of humanized anti-HER2 monoclonal antibody in women who have HER2 overexpressing metastatic breast cancer that has progressed after chemotherapy for metastatic disease. J Clin Oncol 17: 2639–2648 10561337 10.1200/JCO.1999.17.9.2639

[bib7] Dandachi N, Dietze O, Hauser-Krongberg C (2004) Evaluation of the clinical significance of HER-2 amplification by chromogenic *in situ* hybridization in patients with primary breast cancer. Anticancer Res 24: 2401–2406 15330190

[bib8] Dowsett M, Gutierrez C, Mohsin S, Schiff R, Detre S, Johnston S, Osborne CK (2003) Molecular changes in tamoxifen-relapsed breast cancer: relationship between ER, HER2, and p38 MAP-Kinase. Proc Am Soc of Clin Oncol 22: 3 (Abstract 7) 10.1200/JCO.2005.01.172 15753463

[bib9] Edgerton SE, Merkel D, Moore DH, Thor AD (2000) HER-2/neu/erB-b2 status by immunohistochemistry and FISH: clonality and progression with recurrence and metastases. Breast Cancer Res Treat 64: 55

[bib10] Eiermann W (2001) The International Trastuzumab Study Group. Trastuzumab combined with chemotherapy for the treatment of HER-2 positive metastatic breast cancer: pivotal trial data. Ann Oncol 12(Suppl 1): 57–62 11521723

[bib11] Elledge R, Green S, Ciocca D, Pugh R, Allred DC, Clark GM, Hill J, Ravdin P, O’Sullivan J, Martino S, Osborne CK (1998) HER-2 expression and response to tamoxifen in estrogen receptor-positive breast cancer: a Southwest Oncology Group Study. Clin Cancer Res 4: 7–12 9516946

[bib12] Gancberg D, Di Leo A, Cardoso F, Rouas G, Pedrocchi M, Paesmans M, Verhest A, Bernard-Marty C, Piccart MJ, Larsimont D (2002) Comparison of HER-2 status between primary breast cancer and corresponding distant metastatic sites. Ann Oncol 13: 1036–1043 12176781 10.1093/annonc/mdf252

[bib13] Hamilton A, Piccart M (2000) The contribution of molecular markers to the prediction of response in the treatment of breast cancer: a review of the literature on HER-2, p53 and BCL-2. Ann Oncol 11: 647–663 10942052 10.1023/a:1008390429428

[bib14] Harris J, Morrow M, Norton L (1997) Malignant tumors of the breast. In Cancer: Principles and Practice of Oncology, DeVita Jr VT, Hellman S, Rosenberg SA (eds), 5th edn pp 1557–1616. Philadelphia: Lippincott-Raven

[bib15] Hortobagyi G (2001) Optimal duration therapy with trastuzumab. Semin Oncol 28(Suppl 18): 1–2 10.1016/s0093-7754(01)90280-5 11706394

[bib16] Kuukasjarvi T, Karhu R, Tanner M, Kahkonen M, Schaffer A, Nupponen N, Pennanen S, Kallioniemi A, Kallioniemi OP, Isola J (1997) Genetic heterogeneity and clonal evolution underlying development of asynchronous metastasis in human breast cancer. Cancer Res 57: 1597–1604 9108466

[bib17] Leitzel K, Teramoto Y, Konrad K, Chinchilli VM, Volas G, Grossberg H, Harvey H, Demers L, Lipton A (1995) Elevated serum c-erb-B2 antigen levels and decreased response to hormone therapy of breast cancer. J Clin Oncol 13: 1129–1135 7738618 10.1200/JCO.1995.13.5.1129

[bib18] Lipton A, Leitzel K, Ali SM, Demers L, Harvey HA, Chaudri-Ross HA, Lang R, Hackl W, Hamer P, Carney W (2003) Serum HER-2/neu conversion to positive at the time of cancer progression in metastatic breast patients treated with letrozole *vs* tamoxifen. Proc Am Soc Clin Oncol 22: 3 (Abstract 8)

[bib19] Lohrisch C, Piccart M (2001) An overview of HER-2. Semin Oncol 28(Suppl 18): 3–11 11774200

[bib20] Luftner D, Dilk H, Henschke P, Geppert R, Dietel M, Stein H, Wernecke K, Possinger K, Heine P (2004) Results of a 10-year retrospective search in two university institutes of pathology: Concordance of HER-2/neu expression of primary breast carcinomas and their metachronous distant metastases. Proc Am Soc Clin Oncol 23(14S): 44 (Abstract 670)

[bib21] Masood S, Bui MM (2000) Assessment of HER-2/neu overexpression in primary breast cancers and their metastatic lesions: an immunohistochemical study. Ann Clin Lab Sci 30: 259–265 10945565

[bib22] Nicholson RI, McClelland RA, Finlay P, Eaton CL, Gullick WJ, Dixon AR, Robertson JF, Ellis IO, Blamey RW (1993) Relationship between EGF-R, c-erbB-2 protein expression and Ki67 immunostaining in breast cancer and hormone sensitivity. Eur J Cancer 29A: 1018–1023 8098946 10.1016/s0959-8049(05)80215-1

[bib23] Niehans GA, Singleton TP, Dykoski D, Kiang DT (1993) Stability of HER-2/neu expression over time and at multiple metastatic sites. J Natl Cancer Inst 85: 1230–1235 8101229 10.1093/jnci/85.15.1230

[bib24] No author listed (1999) Detection of HER2/neu (c-erbB-2) Antigen Overexpression: An NCI Symposium. Bethesda, MD: Lippincott, October 7–8

[bib25] Paik S, Bryant J, Park C, Fisher B, Tan-Chiu E, Hyams D, Fisher ER, Lippman ME, Wickerham DL, Wolmark N (1998) erb-B2 and response to doxorubicin in patients with axillary lymph node positive, hormone receptor-negative breast cancer. J Natl Cancer Inst 90: 1361–1370 9747867 10.1093/jnci/90.18.1361

[bib26] Ross JS, Fletcher JA (1998) The HER-2/neu oncogene in breast cancer: prognostic factor, predictive factor, and target for therapy. Oncologist 3: 237–252 10388110

[bib27] Shimizu C, Fukutomi T, Tsuda H, Akashi-Tanaka S, Watanabe T, Nanasawa T, Sugihara K (2000) c-erbB-2 protein overexpression and p53 immunoreaction in primary and recurrent breast cancer tissues. J Surg Oncol 73: 17–20 10649273 10.1002/(sici)1096-9098(200001)73:1<17::aid-jso5>3.0.co;2-2

[bib28] Slamon DJ, Clark GM, Wong SG, Levin WJ, Ullrich A, McGuire WL (1987) Human breast cancer: correlation of relapse and survival with amplification of the HER-2/neu oncogene. Science 235: 177–182 3798106 10.1126/science.3798106

[bib29] Slamon DJ, Godolphin W, Jones LA, Holt JA, Wong SG, Keith DE, Levin WJ, Stuart SG, Udove J, Ullrich A, Press MF (1989) Studies of the HER-2/neu proto-oncogene in human breast and ovarian cancer. Science 244: 707–712 2470152 10.1126/science.2470152

[bib30] Slichenmyer W, Fry D (2001) Anticancer therapy targeting the Erb B family of receptor tyrosine kinases. Semin Oncol 28: 67–79 10.1016/s0093-7754(01)90284-2 11706398

[bib31] Tanner M, Jarvinen P, Isola J (2001) Amplification of HER-2/neu and topoisomerase II-*α* in primary and metastatic breast cancer. Cancer Res 61: 5345–5348 11454672

[bib32] Teixeira MR, Pandis N, Bardi G, Andersen JA, Mitelman F, Heim S (1995) Clonal heterogeneity in breast cancer: karyotypic comparisons of multiple intra- and extra-tumorous samples from 3 patients. Int J Cancer 63: 63–68 7558454 10.1002/ijc.2910630113

[bib33] Thor AD, Berry DA, Budman DR, Muss HB, Kute T, Henderson IC, Barcos M, Cirrincione C, Edgerton S, Allred C, Norton L, Liu ET (1998) erbB-2, p53 and efficacy of adjuvant therapy in lymph node-positive breast cancer. J Natl Cancer Inst 90: 1346–1360 9747866 10.1093/jnci/90.18.1346

[bib34] Tsuda H, Hirohashi S, Shimosato Y, Hirota T, Tsugane S, Yamamoto H, Miyajima N, Toyoshima K, Yamamoto T, Yokota J, Yoshda T, Skamoto H, Terada M, Sugimura T (1989) Correlation between long term survival in breast cancer patients and amplification of two putative oncogene co-amplification units: hst-1/int-2 and c-erbB-2/ear-1. Cancer Res 49: 3104–3108 2566377

[bib35] Vogel C, Cobleigh M, Tripathy D, Harris L, Fehrenbacher L, Slamon D, Ash M, Novotny W, Stewart S, Shak S (2001) First-line, non-hormonal, treatment of women with HER2 overexpressing metastatic breast cancer with Herceptin (trastuzumab, humanized anti-HER2 antibody). Proc Am Soc Clin Oncol 19: 71a (Abstract 275)

[bib36] Zidan J, Dashkovsky I, Cozacov C, Hadary A, Basher W (2002) Comparison of HER-2 in primary breast cancer and metastatic sites and its effect on treatment of metastatic disease. ESMO 2002 Ann Oncol 13(Suppl 5): 50 (Abstract 179P) 10.1038/sj.bjc.6602738 PMC2361603 16106267

[bib37] Zidan J, Dashkovsky I, Hadary A, Basher W, Cozacov C (2004) Comparison of HER-2 overexpression in primary breast cancer and metastatic sites and its effect on biological targeting therapy of metastatic disease. Proc Am Soc of Clin Oncol 23: 40 (Abstract 655) 10.1038/sj.bjc.6602738 PMC2361603 16106267

